# Salutational

**Published:** 1883-01

**Authors:** Leigh H. Hunt

**Affiliations:** New York


Salutational.—However agreeable, it is far from an easy matter to greet those who are unknown to one personally, even when it is felt that those we would address are friends. Perhaps it would be demanded of us, and with reason, to "state our intentions."

Promises are things to be chary of—deeds suit most of us far better.

We hope to present to our readers each month a succinct account of whatever advances may have been made in medicine and the allied branches to which the Independent Practitioner is devoted. To this end, translations of events chronicled by the French and German medical journals will be made directly the articles are published in those countries. Our first aim, however, will be to place before the profession the practical results of Medicine, Surgery, Obstetrics, Pathology, Dentistry, and Popular Science won for it on this side of the Atlantic by those men who have a right to be heard—nay, whose duty it is to speak concerning the branches wherein they so markedly excel.

The title of the journal affords a fair criterion for our guidance—*Independent* meaning that unprejudiced views shall be taken, and *Practitioner* warning us that though theory and discussion should form a part, they still fall far short of the importance of what is practical in the domain of medicine. It is to the practitioner that we devote the journal, but in so doing clearness will not be sacrificed to mere brevity, nor reason to mere dogmatic statement. Leigh H. Hunt.

New York, Deeember 25, 1882.

